# A fast-growing dengue virus mutant reveals a dual role of STING in response to infection

**DOI:** 10.1098/rsob.220227

**Published:** 2022-12-14

**Authors:** Wy Ching Ng, Swee Sen Kwek, Bo Sun, Meisam Yousefi, Eugenia Z. Ong, Hwee Cheng Tan, Andreas S. Puschnik, Kuan Rong Chan, Yaw Shin Ooi, Eng Eong Ooi

**Affiliations:** ^1^ Programme in Emerging Infectious Diseases, Duke-National University of Singapore Medical School, Singapore 169857, Singapore; ^2^ Viral Research and Experimental Medicine Center, SingHealth Duke-NUS Academic Medical Center, Singapore 169856, Singapore; ^3^ Chan Zuckerberg Biohub, San Francisco, CA 94158, USA; ^4^ Department of Microbiology and Immunology, Yong Loo Lin School of Medicine, National University of Singapore, Singapore 117597, Singapore; ^5^ Saw Swee Hock School of Public Health, National University of Singapore, Singapore 117549, Singapore

**Keywords:** dengue, NS2B, STING, interferon, autophagy

## Abstract

The four dengue viruses (DENVs) have evolved multiple mechanisms to ensure its survival. Among these mechanisms is the ability to regulate its replication rate, which may contribute to avoiding premature immune activation that limit infection dissemination: DENVs associated with dengue epidemics have shown slower replication rate than pre-epidemic strains. Correspondingly, wild-type DENVs replicate more slowly than their clinically attenuated derivatives. To understand how DENVs ‘make haste slowly’, we generated and screened for DENV2 mutants with accelerated replication that also induced high type-I interferon (IFN) expression in infected cells. We chanced upon a single NS2B-I114T amino acid substitution, in an otherwise highly conserved amino acid residue. Accelerated DENV2 replication damaged host DNA as mutant infection was dependent on host DNA damage repair factors, namely RAD21, EID3 and NEK5. DNA damage induced cGAS/STING signalling and activated early type-I IFN response that inhibited infection dissemination. Unexpectedly, STING activation also supported mutant DENV replication in infected cells through STING-induced autophagy. Our findings thus show that DENV NS2B has multi-faceted role in controlling DENV replication rate and immune evasion and suggest that the dual role of STING in supporting virus replication within infected cells but inhibiting infection dissemination could be particularly advantageous for live attenuated vaccine development.

## Graphical abstract

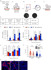

## 1. Introduction

Dengue is an *Aedes* mosquito-transmitted disease that is prevalent throughout the tropical world and is now encroaching into the subtropics [1]. This acute illness, which afflicts an estimated 100 million people each year, some with life-threatening severe dengue, is caused by four antigenically distinct dengue viruses (DENV1–4) [2]. There is no licensed antiviral drug to treat dengue and case management relies entirely on supportive care. A preventative dengue vaccine that can be applied to all, regardless of history of prior exposure to DENV, and with high protective efficacy against all four DENV infection remains an unattained goal. A detailed understanding of dengue pathogenesis is thus urgently needed to enable therapeutics and better vaccines to be successfully developed.

DENV has genome that encodes only 10 genes. It thus relies on interactions with the host cell for successful infection. One of these interactions is the avoidance of premature activation of the innate immune responses, such as type-I interferon (IFN) that restricts infection dissemination and hence likelihood of further virus transmission. While DENV achieves this by inhibiting key innate immune signalling molecules, it could also avoid activating cytoplasmic sensors such as RIG-I by regulating its replication rate. Studies have found that wild-type DENVs and the related yellow fever virus replicate more slowly than their attenuated derivatives [3,4]. In particular, a clinical isolate of DENV2 (16 681 strain) replicated more slowly than its attenuated derivative, DENV2 PDK53 [3,5,6]; DENV2 PDK53 has proven attenuation in several clinical trials [7–9]. Despite the difference in replication rate, DENV2 PDK53 shares an identical polymerase with its wild-type parent, as attenuating mutations were found elsewhere in the genome [5,6]. These observations suggest that, besides polymorphisms in the viral polymerase and replication complex, DENV replication rate may be regulated through virus–host interactions, although such interactions that enable wild-type DENVs to ‘make haste slowly’ remain poorly understood.

Herein, we show how a method that generates and screens for rapidly replicating DENV mutants that are derived from wild-type DENVs could be useful for defining the virus–host interactions that enable DENVs to make haste slowly. We combined 5-fluorouracil (5-FU) chemical mutagenesis, cell sorting, full genome sequencing and infectious clone construction to explore if such a method would be able to identify new genetic variants that induce type-I IFN expression in infected cells. We found a DENV2 mutant from a clinical isolate [10] that possessed only a single I114T amino acid substitution in the NS2B protein. This NS2B-I114T mutant increased dependence of the mutant on DNA repair factors for successful infection to reveal the function of wild-type NS2B in minimizing host nuclear or mitochondrial DNA damage during infection. The DNA damage from mutant infection activated the cGAS/STING pathway. Moreover, this same I114T mutation also impaired the cleavage of STING that would otherwise antagonize type-I IFN induction, the paracrine action of which, expectedly, inhibited virus propagation on a cell monolayer [3]. Unexpectedly, STING activation promoted viral replication in infected cells through increased autophagy—autophagosomes are known to serve as a platform for viral replication complex formation [11]. Our study shows proof-of-concept on a method for discovering rapid replication mutants, reveals the multi-faceted role of NS2B and suggests a hitherto uncertain pro-viral function of the cGAS/STING pathway. The dual role of STING in promoting viral replication while inhibiting infection dissemination could be ideal in promoting replication of attenuated DENV for useful immunogenicity, while limiting systemic infection to ensure clinical safety.

## 2. Results

**Figure 1.**
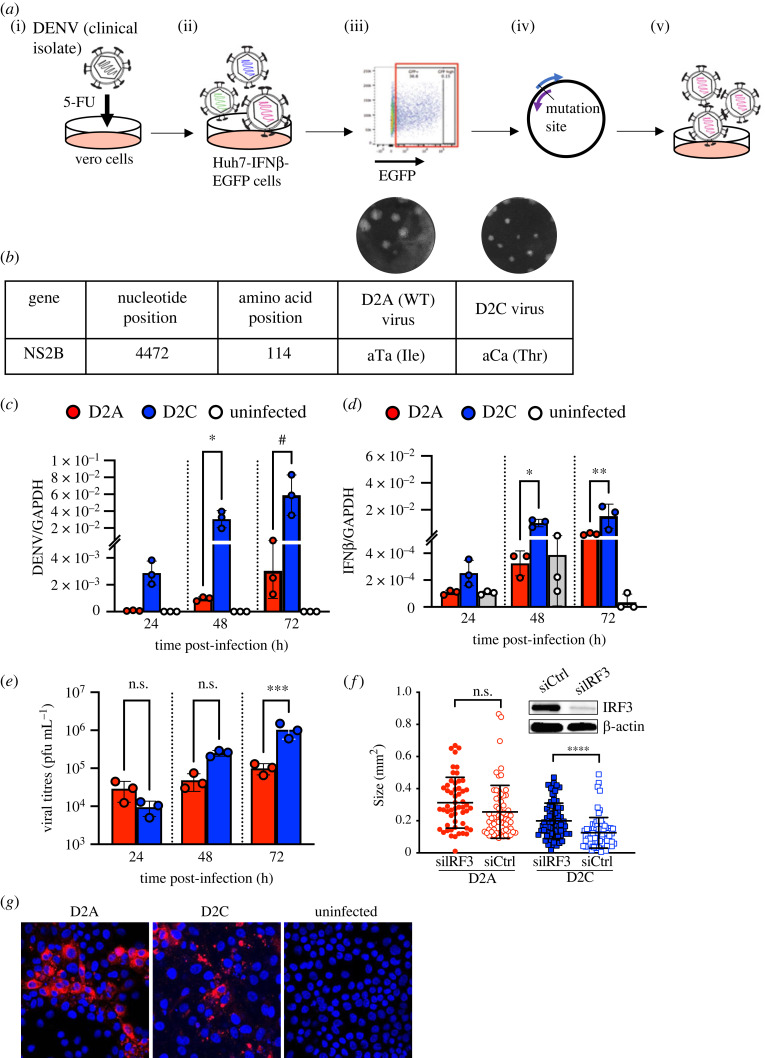
A mutagenesis method produced a single NS2B-I114T mutation that accelerated a clinical isolate of DENV2. (*a*) Schematic diagram of the method. (i) Infection of vero cells in the presence of 5-FU. (ii) HUH-7 cells expressing IFNβ-EGFP promoter infected with supernatant containing heterogeneous population of virus progeny. (iii) Isolation of EGFP-expressing cells by flow cytometry followed by viral RNA extraction for genome sequencing. (iv) Sequencing to ensure that the mutations are successfully engineered into individual fragment plasmids via site-directed mutagenesis. (v) Gibson assembly and amplification of homogeneous population of virus for further characterizations. (*b*) D2C (NS2B-I114T) produced plaques with smaller sizes than D2A (WT) virus. Increased (*c*) DENV RNA and (*d*) IFN*β* mRNA levels in D2C- compared to D2A-infected Huh7 cells 24, 48 and 72 hpi as assessed by qPCR. GAPDH was used as the housekeeping gene for normalization. (*e*) Plaque assay of D2A- and D2C-infected cell supernatants. (*f*) Increased D2C but not D2A plaque size in BHK-21 cells pre-treated with siRNA to silence IRF3. Cropped image shows western blot analysis of cells after 48 h of siRNA transfection to determine IRF3 knockdown efficiency. Beta-actin was used as the housekeeping control. (*g*) Reduced spread of D2C virus 24 hpi observed by immunofluorescence staining of DENV2 envelope protein (red) in Huh7-infected cells. Representative image from D2A, D2C and uninfected cells are shown. Nuclei stained with DAPI (blue). 15 images were taken for each sample. Error bars represent s.d. *p < 0.05, **p < 0.01, ^#^*p* < 0.0001 in one-way ANOVA.

To identify a DENV mutant that induces robust IFN expression shortly following infection, we first developed a Huh7 cell line stably transfected with EGFP under the control of the IFN*β* promoter (Huh7-IFNβ-EGFP). DENV2 (strain D2/SG/05K3295DK1/2005), which was isolated from the blood of a dengue patient [10], was propagated in the presence of a mutagen—5-FU—as previously described [12]. The clarified culture supernatant was then inoculated onto Huh7-IFNβ-EGFP cells. Cell sorting was then used to obtain a pool of Huh7 cells with strong EGFP expression (figure 1*a*). Using full viral genome sequencing and reverse genetics, we were able to rescue a homologous population of virus that contained only a single-nucleotide change, T4472C for further investigation. This nucleotide change corresponded to a isoleucine (I) to threonine (T) amino acid (aa) substitution at position 114 of the NS2B protein (NS2B-I114T) (figure 1*b*). Hereon, we refer to the wild-type DENV2 and the mutant as D2A and D2C, respectively.

Inoculation of D2C onto Huh7 cells showed that this mutant replicated more rapidly than D2A at all post-infection timepoints tested (figure 1*c*). Additionally, D2C infection resulted in significantly elevated IFN*β* expression (figure 1*d*). Similar trends were also observed in primary monocyte-derived dendritic cells (moDCs) (electronic supplementary material, figure S1). Furthermore, secreted IFN*β* levels in infected cell supernatant were significantly elevated with D2C as compared to D2A infection (electronic supplementary material, figure S1). These phenotypes were strikingly similar to the wild-type DENV2 16 681 strain and its attenuated derivative, DENV2 PDK53 [3,6], a candidate live attenuated dengue vaccine [8,13]. DENV2 16681 NS2B has a nucleotide sequence similarity of 93.2% as compared to D2A (electronic supplementary material, figure S2), suggesting that I114T could have attenuated D2C.

Besides elevated D2C genomes, we also observed increased viral progenies in D2C-infected supernatant (figure 1*e*). However, the sizes of D2C plaques were smaller compared to D2A (figure 1*b*). We thus examined if, like DENV2 PDK53, the induced type-I IFN expression was functional in limiting the dissemination of D2C infection on a cell monolayer to result in its reduced plaque size (figure 1*b*). As interferon regulatory factor 3 (IRF3) is the promoter of type-I IFN expression, we silenced IRF3 with siRNA and examined the changes in plaque size. Knockdown of IRF3 significantly increased the plaque size of D2C compared to the control siRNA (figure 1*f*). By contrast, the plaque size of D2A remained unchanged despite IRF3 silencing. These findings indicate that the small plaque size observed in D2C was restricted by IRF3 and IFN*β* upregulation. Moreover, using confocal microscopy, we found reduced dissemination of D2C to uninfected cells within the vicinity of infected cell foci as compared to D2A infection (figure 1*g*). These findings collectively show similarity with the attenuated phenotype of DENV2 PDK53 [3,6].

### 2.1. I114T amino acid substitution is the critical determinant of type-I interferon induction

**Figure 2.**
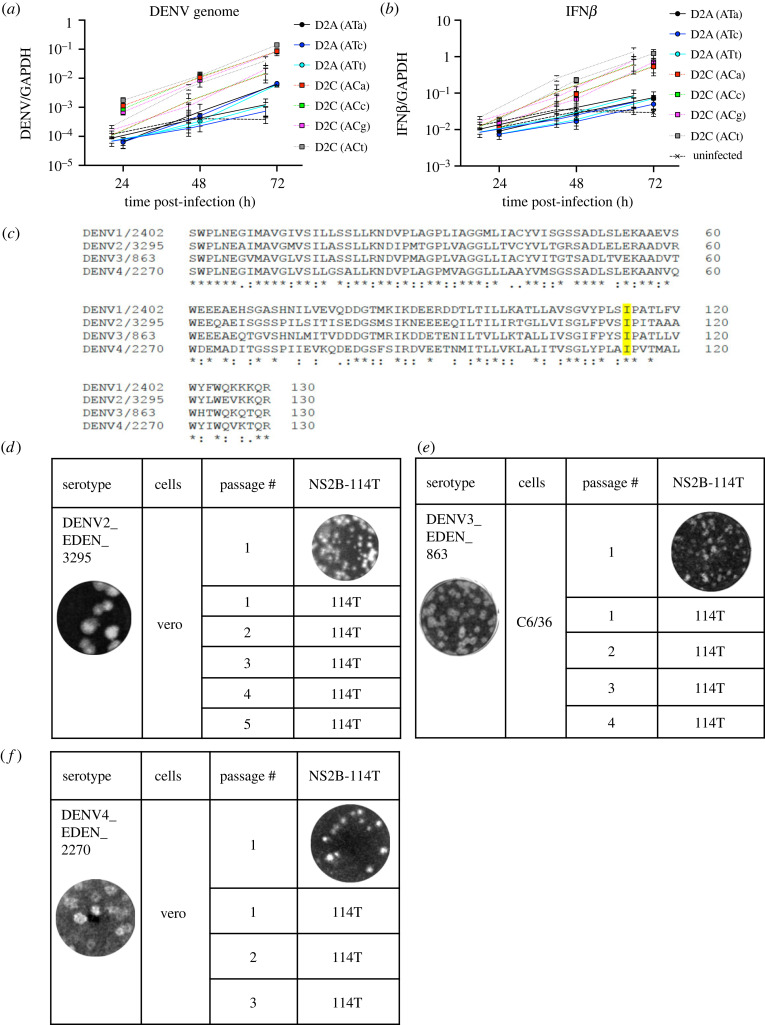
Amino acid isoleucine at position 114 of NS2B induced the D2C phenotype. Increased (*a*) DENV RNA genome and (*b*) IFN*β* mRNA levels at 24, 48 and 72 hpi in D2C-infected Huh7 cells despite nucleotide changes that encode the same amino acid. GAPDH was used as the housekeeping gene for normalization. (*c*) Alignment of NS2B amino acid of all four dengue serotypes. Highlighted region indicates position 114 of NS2B protein. Smaller plaque phenotype of (*d*) DENV2, (*e*) DENV3 and (*f*) DENV4 with engineered NS2B-I114T mutation. Genome stability is shown by serial passaging of virus on Vero (DENV2 and DENV4) or C6/36 (DENV3) cells.

We next investigated whether the change in viral replication and IFN*β* response was caused by substitution of either the nucleotide or amino acid. We thus constructed mutant DENVs with different nucleotide sequences that encode the same amino acid, threonine or isoleucine. We found similarly higher viral RNA replication (figure 2*a*) and IFN*β* response (figure 2*b*) in all nucleotide sequences that coded for threonine as D2C (ACA). This finding excluded the possibility that the observed changes in viral RNA replication and IFN*β* induction were caused by disruption of long-range RNA–RNA interactions [14] in the DENV genome. I114T amino acid substitution thus appeared pivotal for the attenuated phenotype of D2C.

Analysis of the published genome sequences of DENV also showed that isoleucine at position 114 was conserved across all four DENV serotypes (figure 2*c*), raising the possibility that this substitution would also attenuate other DENVs. We thus engineered the NS2B-I114T mutation into DENV3 and DENV4 and found that it produced small plaque phenotype, similar to D2C (figure 2*d–f*). Furthermore, upon passaging of the virus, the mutation was stable and did not revert to wild-type nucleotide after 3 to 5 passages (figure 2*d–f*). The same NS2B-I114T in a DENV1 genome backbone, however, proved unstable and we were unable to obtain a homogeneous population of DENV1 NS2B-I114T.

### 2.2. Host factors critical for D2C viral replication

**Figure 3.**
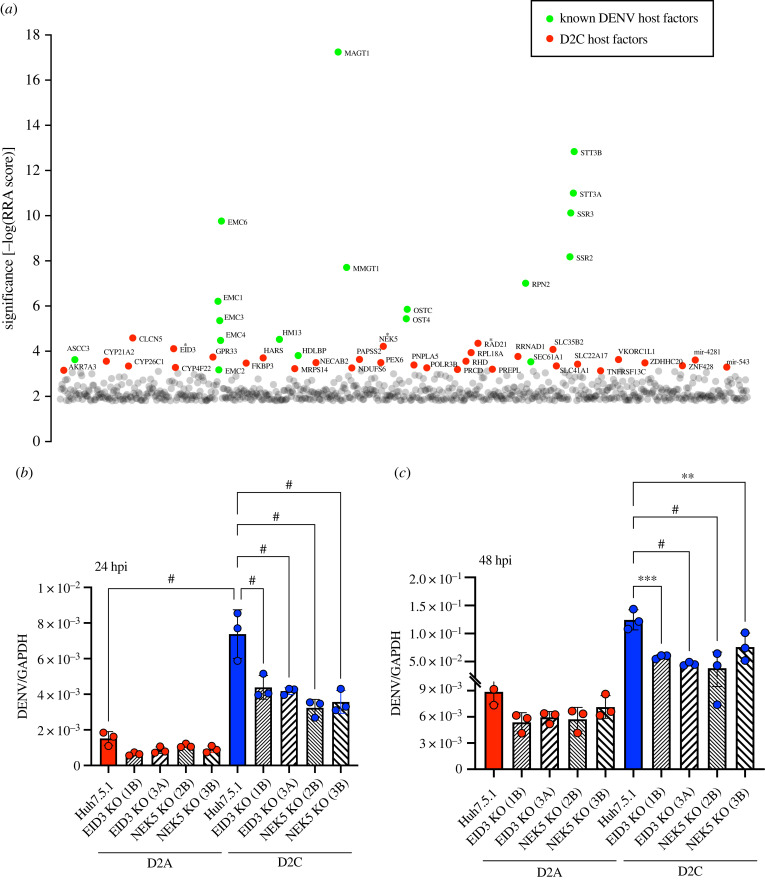
DNA repair genes EID3, NEK5 are essential for D2C replication. (*a*) Gene enrichment for CRISPR screen of D2C infection. Enrichment scores were determined by MaGECK analysis. Significant decrease in D2C viral mRNA levels determined by qPCR in EID3 and NEK5 knockout Huh7.5.1 cells (*b*) 24 and (*c*) 48 hpi. D2A infection unaffected by knockout of EID3 and NEK5. Two gRNAs were selected for each knockout—EID3 1B and 3A; NEK5 2B and 3B. GAPDH was used as the housekeeping gene for normalization. *p < 0.05, **p < 0.01, ^#^*p* < 0.0001 in one-way ANOVA; n.s. not significant. *n* = 2 biological triplicates.

We next examined the mechanism in which I114T elicited the observed phenotype of D2C, which we postulated to be mediated by altered virus–host interactions since this mutation is not in the viral polymerase. To identify how D2C–host interactions could have been altered, we adopted an unbiased approach and conducted a genome-scale CRISPR-Cas9 knockout screen to uncover host factors that were critical for D2C infection in Huh7.5.1 cells. We identified multiple known DENV host factors as top hits, such as multiple components of the oligosaccharyl-transferase complex, several subunits of the endoplasmic reticulum (ER) membrane complex (EMC) and vigilin [15–17], indicating consistency and reproducibility of the functional genomic screening approach. Interestingly, several DNA repair genes, namely RAD21 cohesin complex component (RAD21), E1A-like inhibitor of differentiation 3 (EID3) and NIMA-related kinase 5 (NEK5), were enriched in the D2C but not D2A infection dataset; none of those was previously identified as host factors for any wild-type DENV. This finding suggests that these DNA repair genes could be unique host factors for D2C (figure 3*a*) [15].

Since RAD21 is an essential gene in many cell types [18], it was difficult to obtain 100% knockout of RAD21 genes in cells. Thus, we focused on validating EID3 and NEK5. We generated CRISPR knockout cell populations using two sgRNAs for each gene, respectively. As expected, D2C replication was significantly reduced in EID3 and NEK5 knockout cells compared to the parental Huh7.5.1 cells at both post-infection timepoints tested (figure 3*b,c*). By contrast, D2A replication was unaffected by these two gene knockouts. Our findings suggest that accelerated replication likely caused host nuclear or mitochondrial DNA damage.

### 2.3. NS2B-I114T mutation activated cGAS/STING pathway

**Figure 4.**
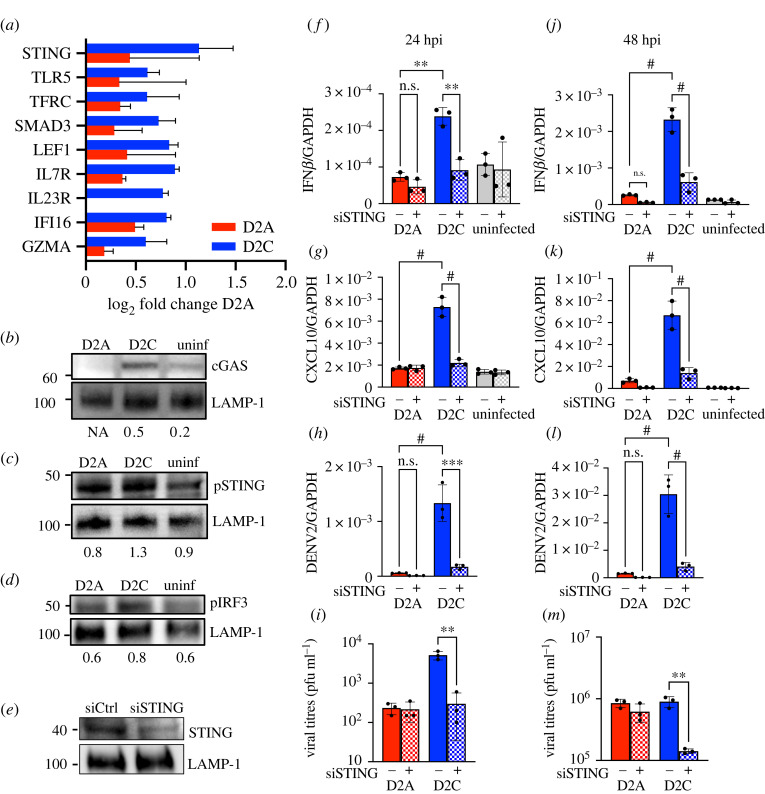
D2C was unable to antagonize the cGAS/STING pathway. (*a*) Increased expression of STING in D2C- compared to D2A-infected moDCs at 24 hpi, as measured by the Nanostring nCounter assay. Values represented as log2-(fold change) compared to uninfected cells. Western blot showing increased (*b*) cGAS protein levels at 24 h, (*c*) pSTING levels at 6 h and (*d*) pIRF3 protein levels at 15 m after infection with D2C virus as compared to D2A or uninfected controls. Values indicate the intensity of the bands after normalization with LAMP-1 as the housekeeping protein, as determined by ImageJ software. One representative image from two blots is shown. (*e*) Western blot analysis of cells after 48 h of siRNA transfection to determine STING knockdown efficiency. Decreased mRNA levels of (*f*,*h*) IFN*β*, (*g*,*i*) CXCL10 and (*j*,*k*) DENV genome in D2C- but not D2A-infected Huh-7 cells (*f*–*h*) 24 and (*j*–*l*) 48 hpi after siRNA knockdown of STING. GAPDH was used as the housekeeping gene for normalization. Plaque assay was performed to show decreased viral titres in D2C-infected supernatant in STING knockdown cells at (*i*) 24 and (*m*) 48 hpi. D2A infection was unaffected by STING knockdown. ^#^*p* < 0.0001 in one-way ANOVA; n.s. not significant. *n* = 2 biological triplicates.

If indeed accelerated D2C replication induced DNA damage, then the type-I IFN expression upon D2C infection could be triggered via the cGAS/STING (cyclic GMA-AMP synthase/stimulator of IFN genes) pathway. The cGAS/STING pathway is known to detect double-stranded DNA which activates downstream effectors to maintain cellular homeostasis. Using a Nanostring nCounter platform to measure the expression of a panel of immune genes in infected moDCs, we found that the most highly upregulated gene in D2C-infected cells was STING (figure 4*a*). Consistently, we also found elevated levels of cGAS (figure 4*b*), as well as the downstream phosphorylated STING (pSTING, figure 4*c*) and IRF3 (pIRF3, figure 4*d*) levels, all by western blots, in D2C but not in either D2A-infected or uninfected cells.

Although D2C infection could have induced DNA damage, the resultant cGAS/STING pathway could nonetheless be inactivated by the known activity of NS2B3 in cleaving human STING [19] and degrading cGAS [20]. It is thus possible that the I114T substitution, besides triggering DNA damage, also altered the interaction of NS2B with STING. As NS2B has previously been shown to inactivate human but not mouse STING [19,21,22], we conducted co-immunoprecipitation assay in Vero cells that overexpressed either mouse or human STING. As expected, both wild-type and mutant NS2B could be co-immunoprecipitated with mouse STING. By contrast, co-immunoprecipitation of wild-type NS2B with human STING was greatly reduced compared to NS2B-I114T (electronic supplementary material, figure S3), consistent with the notion that NS2B3 cleaves STING. In addition, we tested the protease function of WT NS2B3 and NS2B-I114T by engineering the I114T mutation into NS2B3. Both NS2B-NS3pro-flag generated NS3pro product (electronic supplementary material, figure S3). As a negative control, no cleavage product was observed for the inactive NS2B S135A. This finding suggests that the reduced STING cleavage with the NS2B-I114T substitution was likely due to altered NS2B and STING interaction and not impairment of the cleavage activity of NS2B3.

Finally, to show that the wild-type but not mutant NS2B was able to inactivate the function of STING, we examined the impact of STING silencing on viral replication (figure 4*e*). STING knockdown using siRNA resulted in decreased expression of immune genes such as IFN*β* and CXCL10 24 and 48 h (figure 4*f–g*,*j–k*) post-D2C infection. By contrast, immune genes activation was unaffected by STING knockdown after D2A infection. These findings support the role of cGAS/STING in the induction of IFN-related genes upon D2C infection.

### 2.4. STING-induced autophagy increases D2C replication in infected cells

This STING knockdown experiment, unexpectedly, resulted in a significantly reduced D2C replication as evidenced by the lower genome copies and viral progenies at both 24 and 48 hpi in STING knockdown compared to control Huh7 cells (figure 4*h–i*, *l–m*). This observation was surprising since inhibition of type-I IFN induction should have resulted in increased DENV2 infection and replication. This paradoxical finding suggests that although STING-induced type-I IFN response, STING also has pro-viral effects within infected cells.

**Figure 5.**
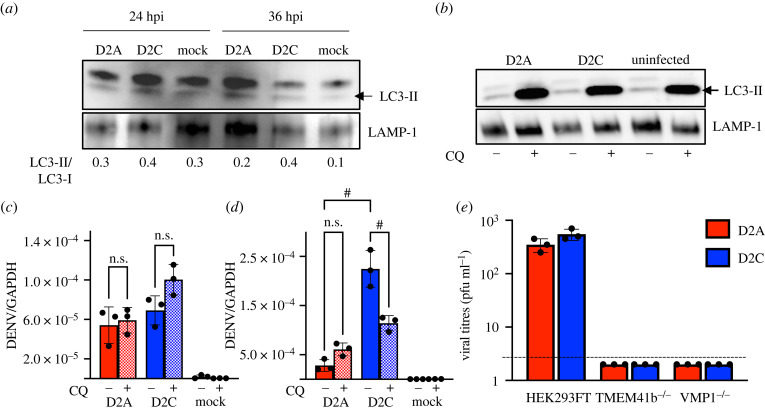
Autophagy upregulation by D2C favoured viral replication. (*a*) Higher ratio of LC3-II/LC3-I in D2C-infected cells as compared to D2A or uninfected cells at 24 and 36 hpi detected by western blot. Quantification of protein was performed with ImageJ software, normalized to LAMP-1. (*b*) Accumulation of LC3-II after treatment with 10 µM of choloroquine (CQ) detected by Western blot. (*c*) Chloroquine treatment did not affect DENV genome mRNA levels 6 hpi. (*d*) Significant reduction of DENV viral genome in D2C-infected Huh7 cells 24 hpi with CQ treatment. (*c*,*d*) Solid bars are infected cells without chloroquine treatment, hatched bars represent cells treated with chloroquine prior to infection. (*e*) The loss of infectious viral particles production in supernatant of TMEM41B and VMP1 knocked out HEK293FT cells. *p < 0.05, ^#^*p* < 0.0001 in one-way ANOVA; n.s. not significant. *n* = 2 biological triplicates.

To explore how STING could mediate a pro-viral effect on D2C infection, we hypothesized that STING promoted D2C replication in infected cells through induction of autophagy. Increased autophagic activity which helps infected cell survive is known to promote flaviviral replication [11,23–25] and STING was recently shown to activate autophagy through a TBK1-independent mechanism [26]. We thus examined whether activation of STING by NS2B-I114T resulted in increased autophagy that then promoted D2C replication in infected cells. First, we detected LC3 via Western blotting as LC3 is used as an autophagosome marker because the amount of LC3-II reflects the number of autophagosomes and autophagy-related structures [27]. We observed a higher ratio of LC3-II/LC3-I at 24 and 36 hpi, suggesting increased autophagy with D2C compared to D2A infection (figure 5*a*). Second, we examined the role of autophagy in DENV2 infection by adding 10 µM of chloroquine 1 h after virus inoculation to inhibit autophagy without inhibiting viral entry. Chloroquine resulted in the accumulation of LC3-II (figure 5*b*) without affecting viral uptake measured at 6 hpi (figure 5*c*). However, at 24 hpi, a decrease in D2C, but not D2A replication was observed (figure 5*d*). Autophagy is critical for DENV infection, as verified by infecting HEK293FT cells that were deficient in autophagosome-modulating genes, TMEM41B and VMP1 [28–30]. Similarly, we found no D2A or D2C progeny produced at 24 hpi when TMEM41B or VMP1 genes were removed (figure 5*e*). Thus, besides inducing IFN response that inhibited virus dissemination, STING activation promoted D2C replication in infected cells.

## 3. Discussion

Avoidance of innate immune responses that limits the total viral burden in its host and hence the likelihood of transmission is central for DENV fitness in its human host. Indeed, besides the differences in replication rate of wild-type DENVs compared to their attenuated derivatives, DENV2 strains isolated during the dengue epidemics have also been found to replicate more slowly than the pre-epidemic strains [31–33]. Despite the possibility that DENVs employ a stealthy replication strategy, exploration of the viral genome landscape for such fitness determinants remain scarce. An approach to identify mutants that impair the ability of DENV to slow its replication and evade host antiviral responses could thus pave the way to explore this poorly understood aspect of viral fitness.

In this study, we devised an experimental approach to derive rapidly replicating DENV2 mutants that also induce a robust IFN response. We combined DENV2 infection with chemical mutagenesis and IFN promoter induced-EGFP screen to recover DENV2 mutants with *in vitro* characteristics similar to the attenuated phenotype of DENV2 PDK53—the formation of small plaques on a plaque assay due to type-I IFN restriction in virus dissemination despite rapid viral RNA replication rate—as previously defined [3,6]. A variation of this approach was also successfully applied to derive an attenuated Zika virus strain [34]. That rapid replication is a feature of reduced clinical and epidemiological fitness is underscored by a DENV strain, namely DENV3 PGMK30/FRhL3 strain, that formed small plaques due to slow replication rate and was proven virulent in humans in clinical trials [3,35]. On the other hand, another DENV strain, DENV1 PDK13, which activated robust innate immune response relative to its wild-type parent, DENV1 16 003, but without a more accelerated replication rate [36] was insufficiently immunogenic in humans [35]; the ability to replicate rapidly before infection is controlled by the innate immune response is important for live attenuated vaccine immunogenicity [37–39]. These findings thus collectively suggest that DENVs that have lost the ability to ‘make haste slowly’ could be useful as live attenuated vaccine candidates. Through these lines of reasoning and our newly devised experimental approach, we identified a single NS2B-I114T substitution that attenuated a clinical isolate of DENV2.

To determine how rapid replication attenuated our mutant, we applied an unbiased genome-wide CRISPR screen that identified DNA repair genes as necessary and unique pro-viral factors for D2C infection. EID3 is part of the SMC5-SMC6 complex which is involved in repair of DNA double-strand breaks by homologous recombination [40]. NEK5 has been shown to be important for DNA damage response, mitochondrial respiration and mtDNA maintenance whereby stable expression of NEK5 resulted in enhanced cell viability [41]. Enrichment of DNA repair genes from D2C infection could thus be indicative of increased mtDNA damage due possibly to its higher replication ability and hence energy consumption. If indeed this postulate is correct, then inability to control host nuclear or mitochondrial DNA damage or increased sensitivity to the consequence of DNA damage may be an underlying mechanism of DENV attenuation.

Although the DENV genome is composed of RNA, the DNA-sensing cGAS/STING pathway is activated upon DENV infection likely through the release of mitochondrial DNA into the cytoplasm [42]. The DNA sensor, cGAS synthesizes cGAMP that binds and activates STING, which mainly localizes to the ER, to form oligomers [43,44]. Following activation, STING then translocates to the Golgi and phosphorylates TBK1 for downstream signal activation [45,46]. Additionally, STING has also been shown to mediate the unfolded protein response thus affecting calcium homeostasis and ER stress [47]. ER stress is known to result in the production of reactive oxygen species (ROS), leading to the upregulation of ISGs [48] as well as other pro-inflammatory responses [49]. Furthermore, ROS can also cause mitochondrial (mt) damage that releases mtDNA, in turn activating the cGAS/STING pathway [42]. STING thus play an important regulatory role in the antiviral response to virus infection. For successful infection, DENV has evolved to curb STING activity whereby the NS2B3 protease cleaves STING to inhibit IFN induction while NS2B degrades cGAS [19,20,22]. Our results thus indicate that NS2B-I114T mutation also resulted in reduced NS2B–STING interaction needed to cleave STING and antagonize the cGAS/STING pathway.

It was previously reported that amino acid (aa) 54–93 of DENV NS2B is part of the core sequence that is required to interact with NS3 for optimal NS2B3 protease activity [50]. The mutation identified in this study, NS2B-I114T lies outside of this region. It is thus likely that this mutation would not alter NS2B interaction with NS3. This mutation is also not within the region of NS2B, aa-75 to −104 and the last 10 aa in the C-terminus, that are critical for interacting with STING [51]. The aa-114 could instead impact the conformation and hence the function of NS2B. It could also affect other interacting partners of STING that we do not yet fully appreciate. For instance, sterol regulatory element-binding cleavage-activating protein, an ER protein was shown to compete with NS2B to bind STING [51]. It is thus possible that the lack of protease activity of NS2B-I114T on STING could also be mediated indirectly, through its interaction with other interacting partners of STING.

While the activation of STING is conventionally associated with antiviral host response, our investigations also suggest a pro-viral property of STING activation mediated through autophagy. Activated STING has been found to be trafficked to the ER Golgi intermediate compartment, which serves as membrane source for LC3 conjugation leading to autophagosome formation [26]. Autophagosome formation is used as a platform for DENV replication complex. They also serve to recruit host triglycerides to increase β-oxidation to enhance ATP production used for viral replication [11,52,53]. Our data showed that NS2B-I114T mutation increased autophagy upon infection and inhibition of autophagy significantly reduced D2C infection. By contrast, IFN*β* response was not affected by autophagy inhibition, suggesting decoupling of the IFN response and activation of autophagy mediated by STING. These findings corroborate previously reported observations where LC3 lipidation can occur independently of TBK1 signalling [26].

How NS2B-I114T accelerated D2C replication is unclear at this stage. It is possible that this mutation altered the conformation of the replication complex that sped up DENV RNA replication. Indeed, DENV2 PDK53 also showed increased rate of RNA replication due to a single NS1-G53D substitution as compared to its parental wild-type DENV2 16 681 [6]; NS1 is also a part of the viral replication complex [54]. However, both proteins are also known to interact with multiple host factors [55,56], suggesting that the D2C phenotype could also be altered by virus–host interactions. Nonetheless, that both NS2B-I114T and NS1-G53D could independently increase RNA replication that attenuate wild-type DENVs further support the notion that slower rate of viral RNA replication enables immune evasion. Indeed, besides the greater risk of activating cytoplasmic RNA sensors, increased RNA replication would consume energy stores more rapidly, which could stress and damage the mitochondria [57,58]. The consequent release of mitochondrial DNA into the cytoplasm could then activate the cGAS/STING pathway and induce type-I IFN response. Perhaps the adage ‘make haste slowly’ applies also to DENV fitness.

In conclusion, our findings suggest the feasibility of a discovery method to generate DENV mutants that are attenuated through accelerated replication and that activation of STING may be a desirable approach to balance DENV replication for both immunogenicity and safety.

## 4. Materials and methods

### 4.1. Cells and virus cultures

BHK-21 (ATCC CCL-10), Vero (ATCC CCL-81), Huh7 (from Duke Cell Repository) and HEK293T (ATCC CRL-3216) were purchased from the American Type Culture Collection (ATCC). Huh7.5.1 was from Frank Chisari, Scripps Research Institute, CA, USA. BHK-21 were cultured in growth media (GM) RPMI 1640 (Gibco); Vero, Huh7, Huh7.5.1 and HEK293T were cultured in DMEM media (Gibco) supplemented with 9% fetal calf serum (FCS, HyClone). Wild-type DENV type 2 strain D2/SG/05K3295DK1/2005 (DENV2, GenBank: EU081177.1), type 3 strain D3/SG/05K863DK1/2005 (DENV3 GenBank: EU081190.1) and type 4 D4/SG/06K2270DK1/2005 (DENV4, GenBank: GQ398256.1) are clinical isolates from the EDEN study [10]. Maintenance media used for infections contain 2% FCS, 100 U ml^−1^ penicillin and 100 µg ml^−1^ streptomycin. All experiments performed with Gibson-assembled DENV were propagated on Vero or C6/36 cells. Viral titres were determined by plaque assay on BHK-21 cells.

Huh7-IFNβ-EGFP cells were constructed by extracting Huh7 genomic DNA using TRIzol LS (Invitrogen). PCR fragments were generated using primer pairs in the electronic supplementary material, table S1 (AseI-IFNB1 Prom and IFNbeta Promoter R) and cloned into pEGFP-C1 plasmid using AseI and NheI restriction enzymes. Transfection of plasmid into Huh7 cells was performed using Lipofectamine 2000 (Thermo Fisher Scientific) as per manufacturer's instructions and cells were maintained in DMEM GM containing 800 µg ml^−1^ Geneticin.

### 4.2. Infectious clone generation and virus propagation

RNA was extracted from DENV using QIAamp Viral RNA Mini Kit (Qiagen) and complementary DNA (cDNA) synthesis was performed using SuperScript III first-strand synthesis Kit (Invitrogen) as per manufacturer's protocols. Six PCR fragments of around 2000 bases long were generated from cDNA using primer pairs in electronic supplementary material, table S1 with NEB Q5 Hot-Start high-fidelity Master Mix (New England Biolabs). Fragments were gel purified using MinElute gel extraction Kit (Qiagen) and TA cloning was performed into pGEM-T Easy Vector (Promega). Plasmids used contain wild-type sequence or required sequence introduced by NEB Q5 Site-Directed Mutagenesis Kit (New England Biolabs) and were sequenced using Sanger sequencing. All six viral genome fragments were amplified using primer pairs in the electronic supplementary material, table S1 with NEB Q5 Hot-Start high-fidelity Master Mix (New England Biolabs). pUC19 vector was also amplified using primer pairs shown in the electronic supplementary material, table S1. Amplified fragments were gel purified and equimolar (0.1 pmole) of each genome fragments and vector were assembled using NEBuilder HiFi DNA Assembly Master Mix (New England Biolabs) at 50°C for 60 m to generate infectious clones. Five microliters of assembled infectious clones was transfected into HEK293T cells in a 24-well tissue culture plate using 3 µL of Lipofectamine 2000 (Thermo Fisher Scientific) as per manufacturer's protocols. Media containing infectious clone-derived virus were collected 72 h post-transfection and passaged on Vero or C6/36 cells in T25 tissue culture flasks to propagate the virus. Virus titres were determined by plaque assay.

### 4.3. Plaque assay

Plaque assay was performed on BHK-21 as previously described [59]. Briefly, serial dilutions (10-fold) of virus were added to BHK-21 cells in 24-well plates and incubated for 1 h at 37°C. Media was aspirated and replaced with 0.9% methyl-cellulose in maintenance media. Five days later, cells were fixed with 20% formalin and stained with 1% crystal violet.

### 4.4. siRNA knockdown of interferon3

BHK-21 cells in 24-well tissue culture plates were transfected with either control small-interfering RNA (siCtrl) or siRNA targeting IRF3 (sense: GGAACAAUGGGAGUUCGAAdTdT and antisense: UUCGAACUCCCAUUGUUCCdTdT) (SABio) using Lipofectamine RNAiMax reagent (Invitrogen) as per manufacturer's protocols. Forty-four post-transfection, plaque assay was performed as described above. Plate was scanned using ImmunoSpot Analyzer (Cellular Technology Ltd.) and smart counting was performed with BioSpot 5.0 software. Transfection efficiency was determined by Western blot using 1:1000 anti-IRF3 (Cell Signaling Technology, #4302S) and 1:1000 anti-β-actin (Cell Signaling Technology, #3700) antibodies.

### 4.5. Monocyte-derived dendritic cells

Peripheral blood mononuclear cells (PBMCs) were isolated from venous blood of a healthy donor. CD14^+^ monocytes were obtained from PBMCs using CD14 microbeads (Miltenyi Biotec) according to manufacturer's protocol. Differentiation of CD14 cells into dendritic cells (moDCs) were done in six-well plates using RPMI 1640 supplemented with 10% FCS, 100 U ml^−1^ penicillin, 100 µg ml^−1^ streptomycin, 100 ng ml^−1^ IL-4 (eBioScience) and 50 ng ml^−1^ granulocyte macrophage-colony stimulating factor (GM-CSF, eBioScience) for 6 days with media change on the third day. moDCs were seeded at 2 × 10^4^ cells per well in 96-well tissue culture plate and infected with DENV2 at multiplicity of infection (MOI) 5 with media change 6 h post-infection. At each time point post-infection, cells were washed once in PBS before lysis in RLT buffer for RNA extraction using QIAamp Viral RNA Mini Kit (Qiagen) according to manufacturer's protocols.

### 4.6. Virus infection

Huh7 cells were seeded at 1 × 10^5^ cells per well in 24-well tissue culture plate 1 day prior to infection. Cells were infected with DENV at MOI of 1 for 1 h before replacement with maintenance media. At various times post-infection, cells were washed once in PBS before lysis in RLT buffer from RNeasy Mini Kit (Qiagen) for RNA extraction according to manufacturer's protocols.

### 4.7. Interferon treatment

Huh7 cells were seeded at 1 × 10^5^ cells per well in 24-well tissue culture plate 1 day prior to infection. Cells were infected at MOI of 1 with or without indicated concentrations of human recombinant IFN (R&D Systems). Supernatant was collected 48 h post-infection and RNA extracted using QIAamp Viral RNA Mini Kit (Qiagen) according to manufacturer's protocols. Viral RNA was quantified using quantitative real-time PCR (qPCR). Per cent inhibition from IFN treatment was quantified relative to infection without IFN treatment.

### 4.8. Nanostring analysis

moDCs were infected with DENVs and total RNA was extracted 24 hpi using the RNeasy Mini Kit (Qiagen). Nanostring profiling of host response was performed using the nCounter Human Immunology v2 Panel. Total RNA (50 ng) was hybridized to reporter and capture probe sets at 65°C for 24 h. Hybridized samples were aligned and immobilized in the nCounter Cartridge and post-hybridization steps and scanning was performed on the nCounter Digital Analyzer (NanoString Technologies). The data (RCC files) was analysed using the nSolver analysis software. Specific genes analyses were done by normalizing counts obtained for the genes to counts for GAPDH. The average log_2_ fold changes normalized to uninfected control. Each sample was performed with biological triplicates.

### 4.9. Quantitative real-time PCR

cDNA from RNA was synthesized using qScript cDNA Synthesis Kit (Quantabio) according to manufacturer's protocols. One microliter of cDNA was used for quantitative real-time PCR performed using LightCycler 480 SYBR Green I (Roche). Gene expression is calculated using the delta–delta Ct method. Primer sequences are listed in electronic supplementary material, table S1.

### 4.10. Western blots

Cell lysates were obtained after incubation in lysis buffer (1% Nonidet P-40, 150 mM NaCl, 50 mM Tris, pH 8.0) supplemented with complete protease inhibitor and resuspended in Laemmli buffer. Crude lysates were boiled for 5 m and then kept on ice. Proteins were separated by SDS-PAGE, transferred to PVDF membrane and incubated with 1 : 1000 rabbit anti-STING (Cell Signaling Technology, #13647S), 1 : 1000 rabbit anti-cGAS (Cell Signaling Technology, #15102S), 1 : 1000 rabbit anti-pSTING 1 : 1000 (Cell Signaling Technology, #85735S), rabbit anti-LC3-II (Cell Signaling Technology, #2775S) or 1 : 3000 mouse anti-LAMP1 (eBioscience, eBioH4A3) followed by 1 : 10 000 HRP-conjugated anti-rabbit or anti-mouse. Blots were developed using ECL™ Prime Western Blotting Detection Reagents.

### 4.11. Flow cytometry

Huh7 cells were infected with D2C virus at MOI 1. 72 h later, cells were harvested and fixed in 80% methanol for 20 m at −20°C. Cells were washed three times with 0.04% BSA in PBS followed by 1 : 1000 mouse anti-NS3 antibody (Genetex) for 1 h at 4°C. Cells were washed three times with 0.04% BSA in PBS followed by 1 : 400 anti-mouse Alexa488 for 30 m at 4°C. Cells were washed and resuspended in FACs buffer before sorting using FACSAria cell sorter (BD Biosciences).

### 4.12. CRISPR-Cas9 genome-wide screen

The genome-scale CRISPR-Cas9 KO screening approach for D2C virus is adapted from [15,60]. Briefly, Cas9 (Addgene #52962, gift from Feng Zhang) was stably introduced into Huh7.5.1 cells followed by transduction of lentiviral sgRNA sub-libraries A and B of the human GeCKO v2 library (Addgene #1000000049, gift from Feng Zhang) [61]. The sgRNA library contained a total of six sgRNA per gene. These transduced cells were selected using puromycin and subsequently expanded for 10 days. A total number of 60 million mutagenized cells for each GeCKO v2 sub-library (A and B) was collected for genomic DNA (gDNA) extraction using Qiagen DNA mini kit to assess the sgRNA representation of the starting population [60], and a total number of 40 million mutagenized cells from each sub-library was infected with D2C virus at MOI of 1 PFU cell^−1^. First sign of cytopathic effects was observed under microscope at 2-day post-infection. A second dose of D2C virus was introduced at MOI of 1 PFU ml^–^^1^ at 5-day post-first inoculation of D2C virus. Massive cell deaths caused by cytolytic infection of D2C virus (greater than 90% of cells are lysed) peaked at 9-day post-first inoculation of the virus, and resistant cells were allowed to proliferate until 18-day post-first inoculation of D2C virus. Approximately, 2 million resistant cells were harvested from each sub-library and subjected to total genomic DNA extraction using DNA mini kit (Qiagen) according to the manufacturer's instructions. The sgRNA were amplified from gDNA in two-step PCR using Herculase II Fusion DNA Polymerase (Agilent). For first PCR, 20 µg of gDNA for each library was amplified for 16 cycles. For second PCR, one reaction containing 5 µl PCR product for each sub-library was amplified for 27 cycles, using Illumina indexed primers. PCR products were gel purified (Qiagen gel purification kit) and sequenced on Illumina MiSeq platform (Duke-NUS Genome Biology Facility) using custom sequencing primers [15]. FASTQ files (ArrayExpress E-MTAB-11119) were analysed using MAGeCK and sgRNA enrichment scores, and gene rankings were determined based on RRA algorithm [62]. FASTQ files for uninfected control are deposited at ArrayExpress E-MTAB-9638 [15].

### 4.13. Generating knockout cell lines

To generate EID3 and NEK5 knockout HUH7.5.1 cells, the following CRISPR sgRNAs obtained from the GeCKO v2 library were cloned into LentiCRISPRV2 plasmid (Addgene, Plasmid #52961) as previously described [61].

gRNA: Sequence
NEK5_2B: GCCTTCTTCAATTCATTTCA
NEK5_3B: ACCTTGAAATGAATTGAAGA
EID_3A: GCGGCACTTCTCCTCGTCAG
EID_1B: ACATTCATCGGCTCTAATA

The cloning product was transfected together with pMDLg/pRRE (Addgene #12251), pMD2.G (Addgene #12259), pRSV-Rev (Addgene #12253) and pAdVantage (Promega E1711) using transIT-LT1 transfection reagent (Mirus Bio) to produce lentivirus in 293FT cells. In parallel, an empty LentiCRISPRV2 plasmid was used to package lentivirus that served as a control. At 48 h post-transfection, lentivirus was harvested from the supernatant and unwanted debris was removed by centrifugation at 500xg for 10 min. One × protamine sulfate (Sigma) was added to the lentivirus before subjecting to transduce Huh7.5.1 cells. Transduced cells were selected by introducing 5 µg ml^−1^ Puromycin (Invivogen) for 4 days.

### 4.14. Statistics

Graphs shown were plotted using GraphPad Prism software. As indicated, one-way ANOVA or two-tailed unpaired Student's *t*-test was used to determine significant differences (*p* < 0.05). Error bars in graphs represent standard deviation with * representing *p* < 0.05, ** representing *p* < 0.01, *** representing *p* < 0.001 and ^#^ representing *p* < 0.0001.

## Data Availability

The data are provided in the electronic supplementary material [63].

## References

[1] Messina JP et al. 2019 The current and future global distribution and population at risk of dengue. Nat. Microbiol. **4**, 1508-1515. (10.1038/s41564-019-0476-8) 31182801 PMC6784886

[2] Bhatt S et al. 2013 The global distribution and burden of dengue. Nature **496**, 504-507. (10.1038/nature12060) 23563266 PMC3651993

[3] Goh KC et al. 2016 Molecular determinants of plaque size as an indicator of dengue virus attenuation. Sci. Rep. **6**, 26100. (10.1038/srep26100) 27185466 PMC4868997

[4] Cong Y, McArthur MA, Cohen M, Jahrling PB, Janosko KB, Josleyn N, Kang K, Zhang T, Holbrook MR. 2016 Characterization of yellow fever virus infection of human and non-human primate antigen presenting cells and their interaction with CD4+ T cells. PLoS Negl. Trop. Dis. **10**, e0004709. (10.1371/journal.pntd.0004709) 27191161 PMC4871483

[5] Butrapet S, Huang CY, Pierro DJ, Bhamarapravati N, Gubler DJ, Kinney RM. 2000 Attenuation markers of a candidate dengue type 2 vaccine virus, strain 16681 (PDK-53), are defined by mutations in the 5’ noncoding region and nonstructural proteins 1 and 3. J. Virol. **74**, 3011-3019. (10.1128/JVI.74.7.3011-3019.2000) 10708415 PMC111799

[6] Choy MM et al. 2020 A non-structural 1 protein G53D substitution attenuates a clinically tested live dengue vaccine. Cell Rep. **31**, 107617. (10.1016/j.celrep.2020.107617) 32402284

[7] Bhamarapravati N, Yoksan S, Chayaniyayothin T, Angsubphakorn S, Bunyaratvej A. 1987 Immunization with a live attenuated dengue-2-virus candidate vaccine (16681-PDK 53): clinical, immunological and biological responses in adult volunteers. Bull World Health Organ. **65**, 189-195. 3496985 PMC2490836

[8] Biswal S et al. 2019 Efficacy of a tetravalent dengue vaccine in healthy children and adolescents. N. Engl. J. Med. **381**, 2009-2019. (10.1056/NEJMoa1903869) 31693803

[9] Saez-Llorens X et al. 2018 Immunogenicity and safety of one versus two doses of tetravalent dengue vaccine in healthy children aged 2–17 years in Asia and Latin America: 18-month interim data from a phase 2, randomised, placebo-controlled study. Lancet Infect. Dis. **18**, 162-170. (10.1016/S1473-3099(17)30632-1) 29122463

[10] Low JG et al. 2006 Early Dengue infection and outcome study (EDEN) - study design and preliminary findings. Ann. Acad. Med. Singapore **35**, 783-789. 17160194

[11] Lee YR et al. 2008 Autophagic machinery activated by dengue virus enhances virus replication. Virology **374**, 240-248. (10.1016/j.virol.2008.02.016) 18353420 PMC7103294

[12] Blaney Jr JE, Johnson DH, Firestone CY, Hanson CT, Murphy BR, Whitehead SS. 2001 Chemical mutagenesis of dengue virus type 4 yields mutant viruses which are temperature sensitive in vero cells or human liver cells and attenuated in mice. J. Virol. **75**, 9731-9740. (10.1128/JVI.75.20.9731-9740.2001) 11559806 PMC114545

[13] Biswal S et al. 2020 Efficacy of a tetravalent dengue vaccine in healthy children aged 4–16 years: a randomised, placebo-controlled, phase 3 trial. Lancet **395**, 1423-1433. (10.1016/S0140-6736(20)30414-1) 32197105

[14] Huber RG et al. 2019 Structure mapping of dengue and Zika viruses reveals functional long-range interactions. Nat. Commun. **10**, 1408. (10.1038/s41467-019-09391-8) 30926818 PMC6441010

[15] Marceau CD et al. 2016 Genetic dissection of Flaviviridae host factors through genome-scale CRISPR screens. Nature **535**, 159-163. (10.1038/nature18631) 27383987 PMC4964798

[16] Ngo AM, Shurtleff MJ, Popova KD, Kulsuptrakul J, Weissman JS, Puschnik AS. 2019 The ER membrane protein complex is required to ensure correct topology and stable expression of flavivirus polyproteins. Elife **8**, e48469. (10.7554/eLife.48469) 31516121 PMC6756788

[17] Ooi YS et al. 2019 An RNA-centric dissection of host complexes controlling flavivirus infection. Nat. Microbiol. **4**, 2369-2382. (10.1038/s41564-019-0518-2) 31384002 PMC6879806

[18] Broad Institute. 2022 CRISPR (DepMap 22Q2 Public Score, Chronos). See https://depmap.org/portal/gene/RAD21?tab=dependency&characterization=expression.

[19] Aguirre S et al. 2012 DENV inhibits type I IFN production in infected cells by cleaving human STING. PLoS Pathog. **8**, e1002934. (10.1371/journal.ppat.1002934) 23055924 PMC3464218

[20] Aguirre S et al. 2017 Dengue virus NS2B protein targets cGAS for degradation and prevents mitochondrial DNA sensing during infection. Nat. Microbiol. **2**, 17037. (10.1038/nmicrobiol.2017.37) 28346446 PMC7457382

[21] Ding Q, Gaska JM, Douam F, Wei L, Kim D, Balev M, Heller B, Ploss A. 2018 Species-specific disruption of STING-dependent antiviral cellular defenses by the Zika virus NS2B3 protease. Proc. Natl Acad. Sci. USA **115**, E6310-E6318. (10.1073/pnas.1803406115) 29915078 PMC6142274

[22] Yu CY, Chang TH, Liang JJ, Chiang RL, Lee YL, Liao CL Lin YL. 2012 Dengue virus targets the adaptor protein MITA to subvert host innate immunity. PLoS Pathog. **8**, e1002780. (10.1371/journal.ppat.1002780) 22761576 PMC3386177

[23] Shrivastava S, Bhanja Chowdhury J, Steele R, Ray R, Ray RB. 2012 Hepatitis C virus upregulates Beclin1 for induction of autophagy and activates mTOR signaling. J. Virol. **86**, 8705-8712. (10.1128/JVI.00616-12) 22674982 PMC3421755

[24] Avirutnan P, Malasit P, Seliger B, Bhakdi S, Husmann M. 1998 Dengue virus infection of human endothelial cells leads to chemokine production, complement activation, and apoptosis. J. Immunol. **161**, 6338-6346. 9834124

[25] McLean JE, Wudzinska A, Datan E, Quaglino D, Zakeri Z. 2011 Flavivirus NS4A-induced autophagy protects cells against death and enhances virus replication. J. Biol. Chem. **286**, 22 147-22 159. (10.1074/jbc.M110.192500) PMC3121359 21511946

[26] Gui X, Yang H, Li T, Tan X, Shi P, Li M Du F, Chen ZJ. 2019 Autophagy induction via STING trafficking is a primordial function of the cGAS pathway. Nature **567**, 262-266. (10.1038/s41586-019-1006-9) 30842662 PMC9417302

[27] Yoshii SR, Mizushima N. 2017 Monitoring and measuring autophagy. Int. J. Mol. Sci. **18**, 1865. (10.3390/ijms18091865) 28846632 PMC5618514

[28] Morita K et al. 2018 Genome-wide CRISPR screen identifies. J. Cell Biol. **217**, 3817-3828. (10.1083/jcb.201804132) 30093494 PMC6219718

[29] Moretti F et al. 2018 TMEM41B is a novel regulator of autophagy and lipid mobilization. EMBO Rep. **19**, e45889. (10.15252/embr.201845889) 30126924 PMC6123663

[30] Yousefi M et al. 2022 TMEM41B and VMP1 modulate cellular lipid and energy metabolism for facilitating dengue virus infection. PLoS Pathog. **18**, e1010763. (10.1371/journal.ppat.1010763) 35939522 PMC9387935

[31] Manokaran G et al. 2015 Dengue subgenomic RNA binds TRIM25 to inhibit interferon expression for epidemiological fitness. Science **350**, 217-221. (10.1126/science.aab3369) 26138103 PMC4824004

[32] Pompon J et al. 2017 Dengue subgenomic flaviviral RNA disrupts immunity in mosquito salivary glands to increase virus transmission. PLoS Pathog. **13**, e1006535. (10.1371/journal.ppat.1006535) 28753642 PMC5555716

[33] Syenina A, Vijaykrishna D, Gan ES, Tan HC, Choy MM, Siriphanitchakorn T Cheng C, Vasudevan SG, Ooi EE. 2020 Positive epistasis between viral polymerase and the 3′ untranslated region of its genome reveals the epidemiologic fitness of dengue virus. Proc. Natl Acad. Sci. USA **117**, 11 038-11 047. (10.1073/pnas.1919287117) PMC7245076 32366663

[34] Kwek SS et al. 2018 A systematic approach to the development of a safe live attenuated Zika vaccine. Nat. Commun. **9**, 1031. (10.1038/s41467-018-03337-2) 29531213 PMC5847552

[35] Kanesa-thasan N et al. 2001 Safety and immunogenicity of attenuated dengue virus vaccines (Aventis Pasteur) in human volunteers. Vaccine **19**, 3179-3188. (10.1016/S0264-410X(01)00020-2) 11312014

[36] Sessions OM, Tan Y, Goh KC, Liu Y, Tan P, Rozen S Ooi EE. 2013 Host cell transcriptome profile during wild-type and attenuated dengue virus infection. PLoS Negl. Trop. Dis. **7**, e2107. (10.1371/journal.pntd.0002107) 23516652 PMC3597485

[37] Chan KR et al. 2016 Cross-reactive antibodies enhance live attenuated virus infection for increased immunogenicity. Nat. Microbiol. **1**, 16164. (10.1038/nmicrobiol.2016.164) 27642668 PMC7097525

[38] Torresi J et al. 2017 Replication and excretion of the live attenuated tetravalent dengue vaccine CYD-TDV in a flavivirus-naive adult population: assessment of vaccine viremia and virus shedding. J. Infect. Dis. **216**, 834-841. (10.1093/infdis/jix314) 28968794

[39] Moodie Z et al. 2018 Neutralizing antibody correlates analysis of tetravalent dengue vaccine efficacy trials in Asia and Latin America. J. Infect. Dis. **217**, 742-753. (10.1093/infdis/jix609) 29194547 PMC5854020

[40] Hudson JJ et al. 2011 Interactions between the Nse3 and Nse4 components of the SMC5–6 complex identify evolutionarily conserved interactions between MAGE and EID Families. PLoS ONE **6**, e17270. (10.1371/journal.pone.0017270) 21364888 PMC3045436

[41] Melo Hanchuk TD, Papa PF, La Guardia PG, Vercesi AE, Kobarg J. 2015 Nek5 interacts with mitochondrial proteins and interferes negatively in mitochondrial mediated cell death and respiration. Cell Signal. **27**, 1168-1177. (10.1016/j.cellsig.2015.02.021) 25725288

[42] Sun B et al. 2017 Dengue virus activates cGAS through the release of mitochondrial DNA. Sci. Rep. **7**, 3594. (10.1038/s41598-017-03932-1) 28620207 PMC5472572

[43] Shang G, Zhang C, Chen ZJ, Bai XC, Zhang X. 2019 Cryo-EM structures of STING reveal its mechanism of activation by cyclic GMP-AMP. Nature **567**, 389-393. (10.1038/s41586-019-0998-5) 30842659 PMC6859894

[44] Shu C, Yi G, Watts T, Kao CC, Li P. 2012 Structure of STING bound to cyclic di-GMP reveals the mechanism of cyclic dinucleotide recognition by the immune system. Nat. Struct. Mol. Biol. **19**, 722-724. (10.1038/nsmb.2331) 22728658 PMC3392545

[45] Saitoh T et al. 2009 Atg9a controls dsDNA-driven dynamic translocation of STING and the innate immune response. Proc. Natl Acad. Sci. USA **106**, 20 842-20 846. (10.1073/pnas.0911267106) PMC2791563 19926846

[46] Liu S et al. 2015 Phosphorylation of innate immune adaptor proteins MAVS, STING, and TRIF induces IRF3 activation. Science **347**, aaa2630. (10.1126/science.aaa2630) 25636800

[47] Wu J, Chen YJ, Dobbs N, Sakai T, Liou J, Miner JJ Yan N. 2019 STING-mediated disruption of calcium homeostasis chronically activates ER stress and primes T cell death. J. Exp. Med. **216**, 867-883. (10.1084/jem.20182192) 30886058 PMC6446864

[48] Olagnier D et al. 2014 Cellular oxidative stress response controls the antiviral and apoptotic programs in dengue virus-infected dendritic cells. PLoS Pathog. **10**, e1004566. (10.1371/journal.ppat.1004566) 25521078 PMC4270780

[49] Chan KR et al. 2019 Metabolic perturbations and cellular stress underpin susceptibility to symptomatic live-attenuated yellow fever infection. Nat. Med. **25**, 1218-1224. (10.1038/s41591-019-0510-7) 31308506

[50] Niyomrattanakit P, Winoyanuwattikun P, Chanprapaph S, Angsuthanasombat C, Panyim S, Katzenmeier G. 2004 Identification of residues in the dengue virus type 2 NS2B cofactor that are critical for NS3 protease activation. J. Virol. **78**, 13 708-13 716. (10.1128/JVI.78.24.13708-13716.2004) PMC533897 15564480

[51] Liu H et al. 2017 Endoplasmic reticulum protein SCAP inhibits dengue virus NS2B3 protease by suppressing its K27-linked polyubiquitylation. J. Virol. **91**, e02234-16. (10.1128/JVI.02234-16) 28228593 PMC5391462

[52] Welsch S et al. 2009 Composition and three-dimensional architecture of the dengue virus replication and assembly sites. Cell Host Microbe **5**, 365-375. (10.1016/j.chom.2009.03.007) 19380115 PMC7103389

[53] Heaton NS, Randall G. 2011 Dengue virus and autophagy. Viruses **3**, 1332-1341. (10.3390/v3081332) 21994782 PMC3185800

[54] Lescar J, Soh S, Lee LT, Vasudevan SG, Kang C, Lim SP. 2018 The dengue virus replication complex: from RNA replication to protein-protein interactions to evasion of innate immunity. Adv. Exp. Med. Biol. **1062**, 115-129. (10.1007/978-981-10-8727-1_9) 29845529

[55] Hafirassou ML et al. 2018 A global interactome map of the dengue virus NS1 identifies virus restriction and dependency host factors. Cell Rep. **22**, 1364. (10.1016/j.celrep.2018.01.038) 29386121

[56] Shah PS et al. 2018 Comparative Flavivirus-host protein interaction mapping reveals mechanisms of dengue and Zika virus pathogenesis. Cell **175**, 1931-1945.e18. (10.1016/j.cell.2018.11.028) 30550790 PMC6474419

[57] El-Bacha T, Midlej V, Pereira da Silva AP, Silva da Costa L, Benchimol M, Galina A, Da Poian AT. 2007 Mitochondrial and bioenergetic dysfunction in human hepatic cells infected with dengue 2 virus. Biochim. Biophys. Acta **1772**, 1158-1166. (10.1016/j.bbadis.2007.08.003) 17964123

[58] Onomoto K, Onoguchi K, Yoneyama M. 2021 Regulation of RIG-I-like receptor-mediated signaling: interaction between host and viral factors. Cell Mol. Immunol. **18**, 539-555. (10.1038/s41423-020-00602-7) 33462384 PMC7812568

[59] Chan KR, Zhang SL, Tan HC, Chan YK, Chow A, Lim AP, Vasudevan SG, Hanson BJ, Ooi EE. 2011 Ligation of Fc gamma receptor IIB inhibits antibody-dependent enhancement of dengue virus infection. Proc. Natl Acad. Sci. USA **108**, 12 479-12 484. (10.1073/pnas.1106568108) 21746897 PMC3145677

[60] Wang R et al. 2021 Genetic screens identify host factors for SARS-CoV-2 and common cold coronaviruses. Cell **184**, 106-119.e14. (10.1016/j.cell.2020.12.004) 33333024 PMC7723770

[61] Sanjana NE, Shalem O, Zhang F. 2014 Improved vectors and genome-wide libraries for CRISPR screening. Nat. Methods **11**, 783-784. (10.1038/nmeth.3047) 25075903 PMC4486245

[62] Li W et al. 2014 MAGeCK enables robust identification of essential genes from genome-scale CRISPR/Cas9 knockout screens. Genome Biol. **15**, 554. (10.1186/s13059-014-0554-4) 25476604 PMC4290824

[63] Ng WC et al. 2022 A fast-growing dengue virus mutant reveals a dual role of STING in response to infection. Figshare. (10.6084/m9.figshare.c.6342730) PMC9748785 36514984

